# A Novel Cytotoxic Mechanism for Triple-Negative Breast Cancer Cells Induced by the Type II Heat-Labile Enterotoxin LT-IIc through Ganglioside Ligation

**DOI:** 10.3390/toxins16070311

**Published:** 2024-07-11

**Authors:** Natalie D. King-Lyons, Aryana S. Bhati, John C. Hu, Lorrie M. Mandell, Gautam N. Shenoy, Hugh J. Willison, Terry D. Connell

**Affiliations:** 1 Department of Microbiology and Immunology, The Jacobs School of Medicine and Biomedical Sciences, The University at Buffalo, The State University of New York, Buffalo, NY 14203, USA; ndking@buffalo.edu (N.D.K.-L.); asb0056@uah.edu (A.S.B.); lmc29@buffalo.edu (L.M.M.); gautam.shenoy@strandtx.com (G.N.S.); 2 The Witebsky Center for Microbiology and Immunology, The University at Buffalo, The State University of New York, Buffalo, NY 14203, USA; conghu@buffalo.edu; 3 Department of Medicine, Division of Infectious Disease, The Jacobs School of Medicine and Biomedical Sciences, The University at Buffalo, The State University of New York, Buffalo, NY 14203, USA; 4 VA Western New York Healthcare System, Buffalo, NY 14215, USA; 5 Institute of Infection, Immunity and Inflammation, University of Glasgow, Glasgow G12 8TA, UK; hugh.willison@glasgow.ac.uk

**Keywords:** enterotoxin, triple-negative breast cancer, ADP-ribosylation, cAMP, ganglioside

## Abstract

Triple-negative breast cancer (TNBC), which constitutes 10–20 percent of all breast cancers, is aggressive, has high metastatic potential, and carries a poor prognosis due to limited treatment options. LT-IIc, a member of the type II subfamily of ADP-ribosylating—heat-labile enterotoxins that bind to a distinctive set of cell-surface ganglioside receptors—is cytotoxic toward TNBC cell lines, but has no cytotoxic activity for non-transformed breast epithelial cells. Here, primary TNBC cells, isolated from resected human tumors, showed an enhanced cytotoxic response specifically toward LT-IIc, in contrast to other enterotoxins that were tested. MDA-MB-231 cells, a model for TNBC, were used to evaluate potential mechanisms of cytotoxicity by LT-IIc, which induced elevated intracellular cAMP and stimulated the cAMP response element-binding protein (CREB) signaling pathway. To dissect the role of ADP-ribosylation, cAMP induction, and ganglioside ligation in the cytotoxic response, MDA-MB-231 cells were exposed to wild-type LT-IIc, the recombinant B-pentamer of LT-IIc that lacks the ADP-ribosylating A polypeptide, or mutants of LT-IIc with an enzymatically inactivated A1-domain. These experiments revealed that the ADP-ribosyltransferase activity of LT-IIc was nonessential for inducing the lethality of MDA-MB-231 cells. In contrast, a mutant LT-IIc with an altered ganglioside binding activity failed to trigger a cytotoxic response in MDA-MB-231 cells. Furthermore, the pharmacological inhibition of ganglioside expression protected MDA-MB-231 cells from the cytotoxic effects of LT-IIc. These data establish that ganglioside ligation, but not the induction of cAMP production nor ADP-ribosyltransferase activity, is essential to initiating the LT-IIc-dependent cell death of MDA-MB-231 cells. These experiments unveiled previously unknown properties of LT-IIc and gangliosides in signal transduction, offering the potential for the targeted treatment of TNBC, an option that is desperately needed.

## 1. Background

Interest in harnessing bacteria, microorganisms, and microbial products as potential anti-cancer agents traces back to William Coley’s 19th-century discovery that a heat-killed blend of Streptococcus and *Serratia marcescens* showed promise in treating bone and soft tissue sarcomas [1]. Since that time, it has been discovered that certain bacteria have a propensity to accumulate in tumor tissues and can deliver anticancer agents directly to the tumor microenvironment. For example, an attenuated strain of *Salmonella enterica* Serovar Typhimurium has been evaluated in Phase I clinical trials for the treatment of metastatic cancers [2], and intravesical *Mycobacterium bovis* or Bacillus Calmette–Guerin (BCG) is part of current first-line therapy for non-muscle invasive bladder cancer [3]. Bacteria have also been used as efficient platforms for the delivery of anticancer agents to tumors [1,4,5]. *Escherichia coli* and *S. enterica* S. Typhimurium, engineered to express cytolysin A, a bacterial pore-forming hemolytic protein, inhibited tumor growth [6,7]. Cytolethal bacterial toxins including diphtheria toxin, Pseudomonas exotoxin A, and *Clostridium perfringens* CPE toxin have also been explored as potential anticancer agents [8,9,10]. The use of these toxins, however, required developing methods to reduce their toxicity and off-target effects. Exotoxin A was linked to an anti-CD25 antibody to specifically target cells abundant in CD25 on their surfaces, such as those found in adult T-cell leukemia and hairy cell leukemia [10]. Hence, efforts persist in the quest for novel cytolethal microbial compounds that possess an inherent ability to selectively target cancer cells while displaying minimal cytotoxicity towards healthy cells.

Novel therapeutic strategies are urgently needed for cancers that lack defined targets for specific and effective therapy. The treatment of triple-negative breast cancer (TNBC) is particularly challenging, due to its negligible expression of targetable receptors like estrogen receptor (ER), progesterone receptor (PR), and human epidermal growth factor receptor 2 (HER2/Neu/ErbB2) [11,12]. For this reason, TNBC patients are typically treated with chemotherapy and radiation, a strategy that has poor efficacy due to the high presence of cancer stem cells in TNBC [13] and carrying substantial side effects. Cancer stem cells have been implicated in the therapeutic resistance of TNBC due to their ability to evade cell death induced by radiation and standard chemotherapeutics. Although TNBC accounts for only 15–20% of breast cancer, patients diagnosed with TNBC are at a disproportionately higher risk of disease recurrence, progression, and death [14]. Therefore, to provide effective treatment options for women with TNBC, alternate targets of therapy need to be defined.

One promising therapeutic target is the ganglioside subtype preferentially expressed on cancer cells [15,16,17,18]. These cell-surface markers can be targeted using heat-labile enterotoxins (HLT), a family of structurally related proteins that is divided into two major groups. The type I HLTs include CT expressed by *Vibrio cholerae* and LT-I expressed by some enterotoxic strains of *E. coli* [19]. LT-IIa, LT-IIb, and LT-IIc expressed by certain enterotoxic strains of *E. coli* comprise the type II subfamily of HLTs [20,21,22]. Both type I and type II HLTs are oligomeric proteins comprised of a single A polypeptide that is non-covalently bound to a pentameric array of B polypeptides [23,24]. While the A-subunit harbors potent ADP-ribosyltransferase activity in the A1-domain, the B-pentamer has affinity for one or more ganglioside receptors found on the plasma membrane of eukaryotic cells. Upon binding to the cell-surface gangliosides, the HLTs are internalized via endocytosis and trafficked to the Golgi via retrograde transport [25]. The A-subunit of each HLT is delivered to the cytoplasm, wherein the Gs-alpha (Gsα) subunit of the G protein regulatory complex is targeted for ADP-ribosylation. The ADP-ribosylation of Gsα induces the constitutive activation of adenylyl cyclase, which significantly elevates the intracellular concentration of cAMP, a strong secondary messenger [26]. cAMP activates protein kinase A, which phosphorylates the cAMP response element binding protein (CREB), a nuclear transcription factor that regulates a variety of processes including cellular proliferation, differentiation, and survival [27,28]. The enterotoxigenic functions of type I and type II HLTs require the HLTs’ ADP-ribosyltransferase activity to elevate levels of intracellular cAMP [27].

Using MDA-MB-231 cells as a model of TNBC, LT-IIc was shown to induce autophagy while simultaneously blocking autolysosomal progression [29], a response that is predicted to be detrimental to cell viability. Subsequent cytotoxicity assays demonstrated that LT-IIc was indeed cytotoxic for MDA-MB-231 cells and for other triple-negative breast cancer (TNBC) cell lines [29]. Conversely, LT-IIc exhibited little or no cytotoxic activity toward ER+ or Her2+ cancer cell lines or for non-TNBC cell types including MCF10A, an immortalized but non-transformed breast epithelial cell line [29]. Only a brief 1 h exposure to LT-IIc was sufficient to induce cytotoxicity in TNBC cell lines, indicating that the effect of LT-IIc on TNBC cells was rapid and irreversible (TDC and NKL, unpublished data). Further analysis established that LT-IIc induced both apoptosis and necroptosis in TNBC cells [29].

The precise mechanism by which LT-IIc triggers TNBC-specific cytotoxicity, however, has not been defined. Since most of the known activities of type I and type II HLTs are dependent upon the capacity of the enterotoxins to ADP-ribosylate GSα and to increase the intracellular levels of cAMP, it is postulated that one or both of those activities is required for LT-IIc to trigger cytotoxic responses in TNBC cells. This hypothesis was supported by studies in which agents that increased the intracellular cAMP to non-physiological levels suppressed the growth of various types of cells by triggering apoptosis or cell-cycle arrest [30,31,32].

Herein, it is demonstrated that LT-IIc also stimulates a cytotoxic response in primary cells isolated from human resected tumors of the TNBC subtype, supporting the use of TNBC cell lines to delineate the mechanisms underlying the cytotoxic properties of LT-IIc for TNBC cells. Using MDA-MB-231 cells as a TNBC model to evaluate the cytotoxic mechanism of LT-IIc, its capacity to elevate intracellular cAMP or the toxin’s ADP-ribosyltransferase activity were revealed to be dispensable for the cytotoxic response of TNBC cells. The full cytotoxic effect, however, required the presence of the A1-domain, with or without the ADP-ribosyltransferase activity, indicating a novel activity of the LT-IIc A-subunit. Experiments using several LT-IIc mutants, the recombinant B-pentamer of LT-IIc, and pharmacological inhibitors established that triggering of the irreversible cytotoxic response in TNBC cells required the ligation by LT-IIc of one or more types of cell-surface ganglioside receptors. These data indicate a novel role for gangliosides in signal transduction. These data also raise the possibility that engineered anti-ganglioside biologics, such as humanized monoclonal antibodies, directed at those specific gangliosides could serve as novel therapeutics for primary and metastatic TNBC, a type of breast cancer that currently has few treatment options.

## 2. Results

### 2.1. LT-IIc Elicits a Strong Cytotoxic Response in Primary Triple-Negative Breast Cancer (TNBC) Cells

**Figure 1:**
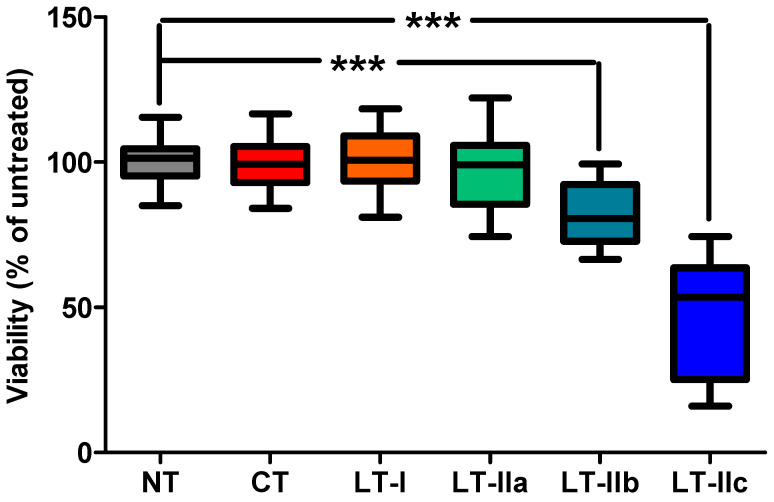
LT-IIc elicits a strong cytotoxic response in primary cancer cells isolated from TNBC tumors. Primary cells, isolated from four human, resected tumors were each treated (in replicates of 6) with 62 nM of type I (CT and LT-I) and type II (LT-IIa, LT-IIb, and LT-IIc) HLTs for 48 h, analyzed for viability by MTT assay, and normalized to the mean absorbance at 570 nM of untreated cells (NT) from the same tumor. Statistical significance was measured by one-way ANOVA followed by Tukey’s multiple comparison test. *** *p* < 0.0001.

Previously, we reported that LT-IIc induced a significant decrease in the viable cell numbers in the TNBC cells lines MDA-MB-231 and BT549 [29]. This cytotoxic effect was also shown to be specific to LT-IIc, as other types of HLTs were unable to stimulate a strong cytotoxic response on MDA-MB-231 cells [29]. Here, primary cells that were isolated from tumors surgically resected from patients diagnosed with TNBC were treated, ex vivo, with either PBS, or with 62 nM (5 μg/mL) of type I (CT and LT-I) or type II (LT-IIa, LT-IIb, and LT-IIc) HLTs. As was the case for TNBC cells lines [29], the viability of primary TNBC cells substantially decreased to an average of 48% (compared to untreated) after treatment with LT-IIc, while little cytotoxic effect was observed after treatment with the other HLTs (Figure 1). Specifically, treatment with CT, LT-I, and LT-IIa had no effect on viability, while treatment with LT-IIb showed a small but significant effect, reducing viable cell numbers to an average of 82% (Figure 1). These data provided evidence that the cytotoxic effect of LT-IIc on TNBC remains relevant in clinical specimens and justified the investigation of the mechanisms driving the cytotoxic activity of LT-IIc. Experiments were designed to evaluate the importance of cAMP induction, GSα ribosylation, and ganglioside ligation, the major activities of these enterotoxins, in the cytotoxic activity of LT-IIc for TNBC cells.

### 2.2. HLTs Stimulate the cAMP Signaling Pathway in MDA-MB-231

**Figure 2:**
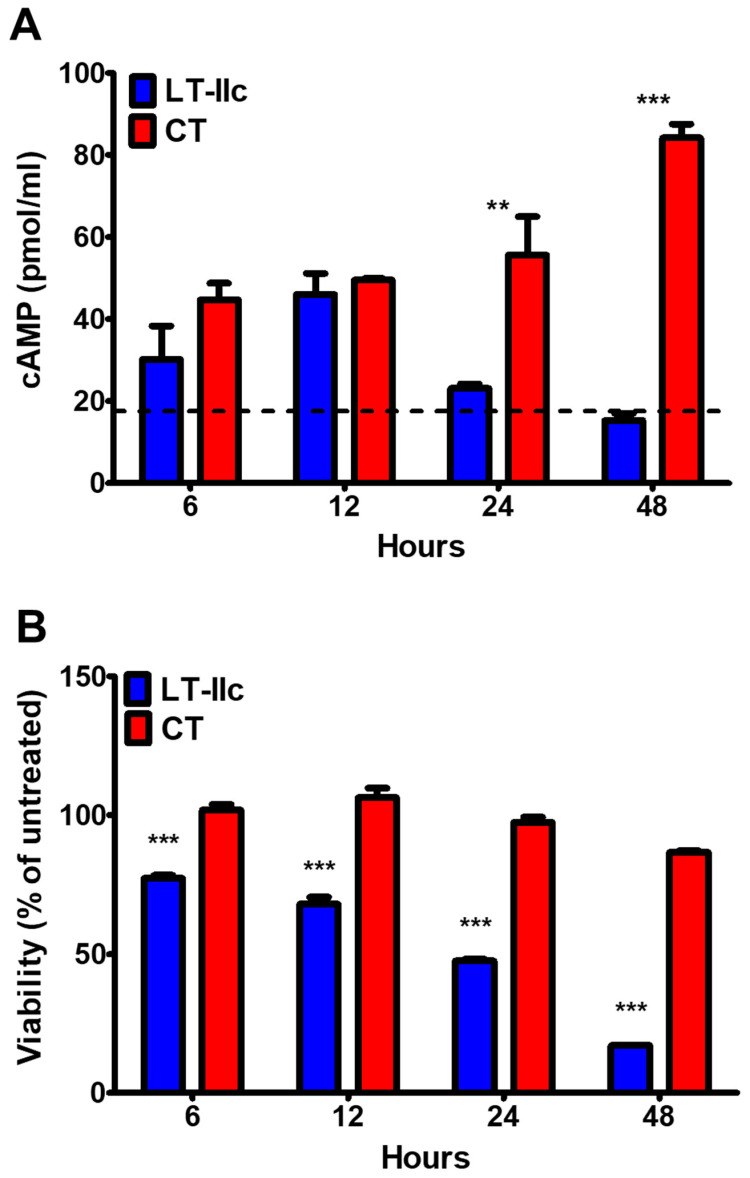
The effects of LT-IIc versus CT A1:B5 holotoxins on cAMP accumulation and viability in MDA-MB-231 cells. MDA-MB-231 cells were treated with 62 nM LT-IIc or CT for 6, 12, 24, or 48 h and were analyzed for (**A**) intracellular cAMP accumulation measured by ELISA (n = 3), with the concentration of cAMP measured in untreated cells indicated by a dashed line; or (**B**) viability compared to untreated cells measured by MTT assay (n = 6). Data points represent mean +/− SEM. Statistical significance was measured by two-way ANOVA. ** *p* <0.01, *** *p* < 0.001.

ADP-ribosylation of GSα by the enzymatic activity of the A polypeptide of LT-IIc constitutively activates adenylyl cyclase in gut epithelial cells [22]. To confirm that the ADP-ribosyltransferase activity of LT-IIc had the same effect on MDA-MB-231 TNBC cells, cells were treated either with 62 nM of LT-IIc or with CT in the holotoxin form (A1:B5). Within 6 h, the concentration of intracellular cAMP in the MDA-MB-231 cells treated with LT-IIc nearly doubled, reaching 30.17 pmol/mL in comparison to the 17.1 pmol/mL observed in the untreated cells. The levels of cAMP attained 46.06 pmol/mL in cells treated for 12 h with LT-IIc (Figure 2A). By 24 h, however, the level of cAMP decreased in the LT-IIc-treated cells to levels only slightly above the concentration observed in the untreated cells. In contrast, the cAMP levels in the CT-treated cells remained elevated, with the concentration of accumulated cAMP in the CT-treated cells increasing over time to levels twice the maximal level observed when cells were treated with LT-IIc (84.15 pmol/mL at 48 h vs. 46.06 pmol/mL at 12 h) (Figure 2A). Thus, both LT-IIc and CT were able to stimulate cAMP production in MDA-MB-231, indicating that both HLTs are internalized by MDA-MB-231 cells and that the ADP-ribosyltransferase containing A-subunits are delivered to the cytoplasm for the ADP-ribosylation of GSα.

### 2.3. LT-IIc-Dependent Induction of Cellular cAMP Is Not Required for Cytotoxicity

MDA-MB-231 cells treated with LT-IIc exhibited a significant decrease in cell viability, as measured by the MTT assay (Figure 2B). Only 48% of the cells remained viable at 24 h, and this percentage was reduced to 17% by 48 h (Figure 2B). This decrease in viable cell count correlates with the decrease in cAMP production in LT-IIc-treated MDA-MB-231 seen in Figure 2A. In contrast, the activation of cAMP production by CT was not accompanied by cytotoxicity. MDA-MB-231 treated with CT was viable after 48 h, with 87% of the cells producing formazan crystals in the MTT assay (Figure 2B). Previously, we demonstrated that MDA-MB-231 cells’ viability was not affected by treating cells with forskolin [29], which activates the enzyme adenylyl cyclase. These data further support that increasing cAMP does not contribute to the cytotoxic response of MDA-MB-231 to LT-IIc.

### 2.4. HLTs Stimulate PKA Downstream Signaling with Distinctive Kinetics

**Figure 3:**
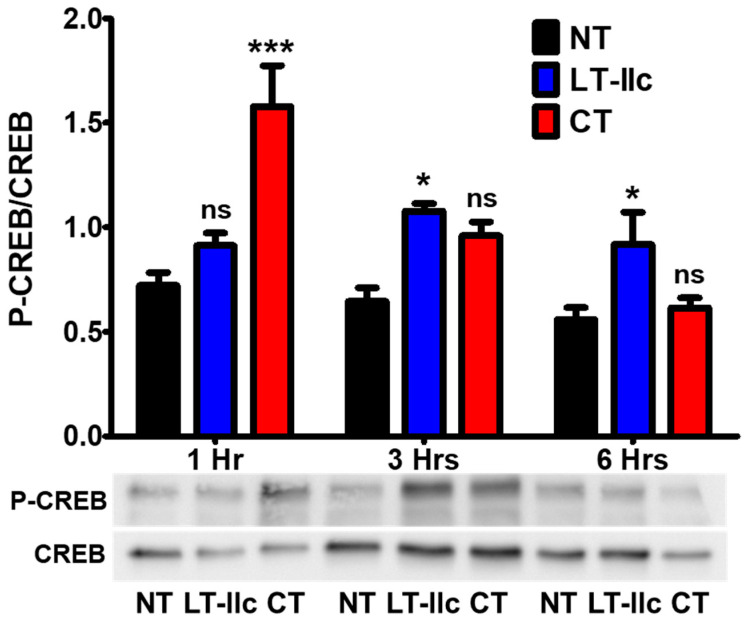
Effects of LT-IIc versus CT A1:B5 holotoxins on CREB phosphorylation. MDA-MB-231 cells were untreated (NT) or treated, in triplicate, with 62 nM LT-IIc or CT for 1, 3, and 6 h. P-CREB and CREB were measured in whole-cell lysates of MDA-MB-231 by immunoblotting. Blots were quantified using ImageJ software (https://imagej.net/ij/). A representative blot is shown below graph of relative quantitation of P-CREB/CREB. Data points represent mean +/− SD. Statistical significance was measured by comparison to untreated cells using two-way ANOVA. * *p* < 0.5; *** *p* < 0.001; ns = not significant.

In many cells, elevation of the intracellular levels of cAMP indirectly controls the phosphorylation of CREB (P-CREB) via the direct activation of PKA to regulate cellular responses including apoptosis [33]. MBA-MB-231 cells that were treated with LT-IIc or CT were analyzed for CREB phosphorylation. After 3 h of exposure to LT-IIc, the P-CREB/CREB ratio in the LT-IIc-treated cells was significantly higher (*p* < 0.01) than the ratio measured in the untreated cells and continued to increase at 6 h (*p* < 0.001) (Figure 3), confirming that LT-IIc modulated the CREB pathway in MDA-MB-231. In contrast, after 1 h of treatment with CT, cells exhibited an increased level of phosphorylation of CREB. This increase, however, was diminished by 3 h (Figure 3). These data suggest that the cAMP-PKA-CREB signaling pathway is functional in MDA-MB-231 cells and responds to both LT-IIc and to CT holotoxins. These data also firmly demonstrate that the increase in intracellular cAMP induced by LT-IIc (and by CT) (Figure 2A) and the phosphorylation of downstream signaling targets (Figure 3) are not intrinsically linked to the cytotoxic response (Figure 2B).

### 2.5. ADP-Ribosylation Activity of LT-IIc Is Not Essential for Cytotoxicity

**Figure 4:**
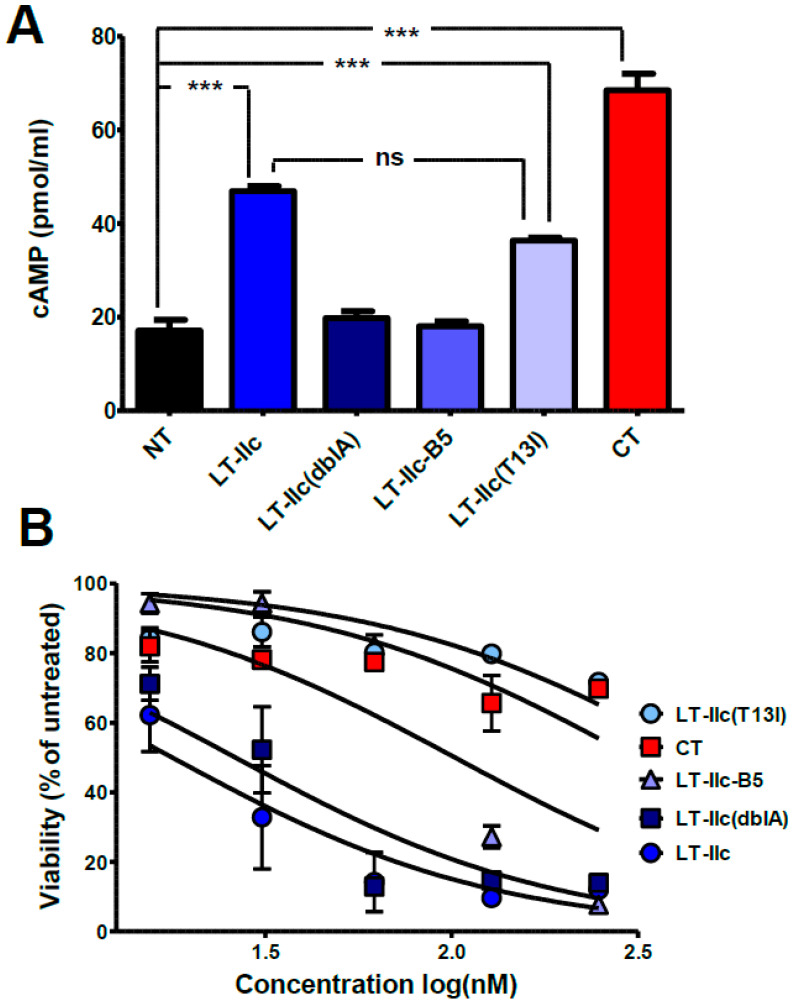
The effects of ADP-ribosylation and the ganglioside binding activity of LT-IIc holotoxin variants versus B5 pentamer on cAMP accumulation and viability in MDA-MB-231 cells. (**A**) MDA-MB-231 cells were untreated (NT) or treated with 62 nM wt LT-IIc, LT-IIc(dblA), LT-IIc-B5, LT-IIc(T13I), or CT for 6 h, and were analyzed for intracellular cAMP accumulation measured by ELISA (n = 3). Statistical significance was measured by one-way ANOVA. *** *p* < 0.001; ns = not significant. (**B**) MDA-MB-231 cells were treated with 0, 15.5, 31, 62, 124, and 248 nM wt LT-IIc, LT-IIc(dblA), LT-IIc-B5, LT-IIc(T13I), or CT for 48 h and viability was measured by MTT assay (n = 4). Data points represent mean +/− SD.

Although the rise in intracellular cAMP, modulated by the ADP-ribosylation of GSα, is not sufficient to induce the cytotoxic effects of LT-IIc (Figure 2A,B), it is possible that alternate targets of ADP-ribosylation, other than GSα, may be required for LT-IIc-dependent cytotoxicity. In previous studies, an ADP-ribosyltransferase inactivating double amino acid substitution (S59K and E108K) was engineered in the A polypeptide of LT-IIb, a type II HLT that is closely related to LT-IIc [34]. To dissect whether ADP-ribosyltransferase activity is required for cytotoxicity of LT-IIc, a similar S59K/E108K mutation was engineered in the A polypeptide of LT-IIc [LT-IIc(dblA)]. As expected, MDA-MB-231 cells treated for 6 h with 62 nM of LT-IIc(dblA) had no significant increase in intracellular cAMP over the levels expressed in untreated cells (Figure 4A). To determine if the ADP-ribosyltransferase activity of LT-IIc was required for cytotoxicity, MDA-MB-231 cells were treated with LT-IIc(dblA) and their viability was determined using an MTT assay. The cytotoxic activity of LT-IIc(dblA), with a calculated IC50 of 26.3 nM, was essentially equivalent to the cytotoxic activity of wt LT-IIc, that has an IC50 of 17.8 nM (Figure 4B). These data clearly demonstrated that the capacity of LT-IIc to kill MDA-MB-231 cells did not require the HLT’s ADP-ribosyltransferase activity.

### 2.6. The A-Subunit of LT-IIc Is Not Necessary for Triggering Cytotoxicity in MDA-MB-231

Although the cytotoxic capacity of LT-IIc was unlinked to the cAMP pathway (via ADP-ribosyltransferase activity on Gsα), or ADP ribosyltransferase activity in general, it was possible that the A-subunit of LT-IIc harbored an unknown activity that modulated the HLT’s cytotoxic activity in TNBC. Recombinant B-pentamers of the type II HLTs, expressed in the absence of the A polypeptide, can be purified and are stable. These B-pentamers have been shown to fully retain the ganglioside-binding properties of the various Type I and II HLTs [35]. To determine if the A polypeptide of LT-IIc had a role in the cytotoxicity, the recombinant LT-IIc B-pentamer (LT-IIc-B5) was engineered, purified, and employed in cAMP and MTT assays. Similar to the effects of LT-IIc(dblA), MDA-MB-231 cells treated with 62 nM LT-IIc-B5 exhibited no significant increase in cAMP accumulation over the levels observed in untreated cells (Figure 4A). MTT assays revealed that LT-IIc-B5 exhibited an attenuated cytotoxic activity for MDA-MB-231 cells. The IC50 for LT-IIc-B5 was calculated to be 102.1 nM, indicating that an approximately four-fold higher concentration of LT-IIc-B5 was required to attain the same level of cytotoxicity as the A1:B5 holotoxins wt LT-IIc or the LT-IIc(dblA) double mutant (Figure 4B). The treatment with 62 nM of holotoxin of either wild-type LT-IIc or LT-IIc(dblA) reduced the number of viable cells to ~14% of the untreated control. At the same concentration, LT-IIc-B5 had little effect on viability, with 82% of the cells remaining metabolically active (Figure 4B). MDA-MB-231 cells, however, did respond when higher concentrations of LT-IIc-B5 were employed. Only 27% of the MDA-MB-231 remained viable when the concentration of LT-IIc-B5 was increased to 124 nM. The viability further decreased to 7% when the concentration of LT-IIc-B5 was increased to 248 nM (Figure 4B).

In contrast to LT-IIc, CT, with an IC50 calculated to be 309.5 nM, had little or no effect on the viability of MDA-MB-231 cells with 70% of the cells maintaining metabolic fitness when treated with 248 nM of CT (Figure 4B). Despite the need for a higher concentration of LT-IIc-B5 to achieve the same level of cytotoxicity as wild-type LT-IIc, these data strongly support the model that the cytotoxic activity of LT-IIc for MDA-MB-231 cells is largely independent of the A-subunit of the HLT, and is instead due to the ganglioside-binding activity of the LT-IIc B-pentamer.

### 2.7. The A1-Domain of LT-IIc Is Required for Full Cytotoxicity

**Figure 5:**
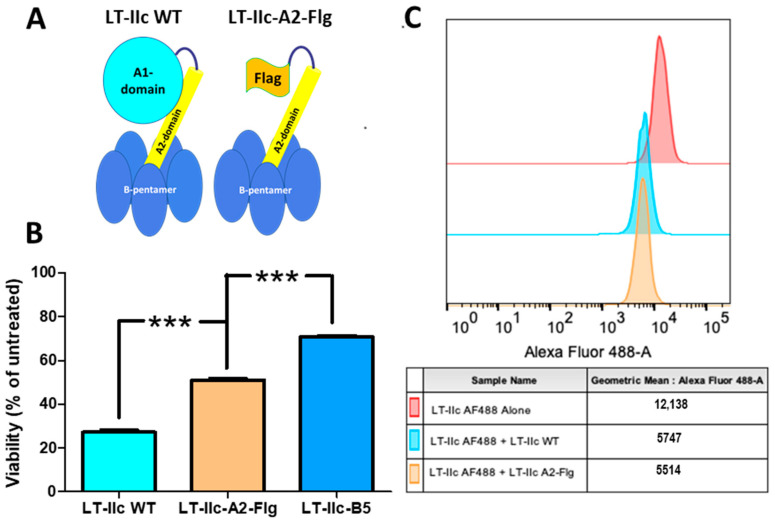
Effects of the A1-domain of LT-IIc on cytotoxicity and binding affinity. (**A**) Cartoon representation of the A1-domain (light blue), A2-domain (yellow), and B-pentamer (blue) of LT-IIc holotoxin and LT-IIc-A2-Flg chimeric protein in which the A1-domain is replaced by a Flag epitope tag (orange). (**B**) MDA-MB-231 cells were treated with 62 nM LT-IIc, LT-IIc-A2-Flg, or LT-IIc-B5 for 48 h and analyzed for viability compared to untreated cells measured by MTT assay (n = 6). Data points represent mean +/− SEM. Statistical significance was measured by one-way ANOVA. *** *p* < 0.0001. (**C**) Example of one of the four flow cytometric analyses measuring the effect of unlabeled LT-IIc (light blue) and unlabeled LT-IIc-A2-Flg (orange) on the mean fluorescent intensity of MDA-MB-231 cells stained with Alexa Fluor 488-labeled LT-IIc (red).

The differences in the cytotoxic profiles between the LT-IIc holotoxin and the B-pentamer suggest a role for the A-subunit apart from the ADP-ribosyltransferase activity in the cytotoxic response of MDA-MB-231 cells. The A-subunit may possess an activity that is unique to LT-IIc, or the A-subunit may provide structural support to the B-pentamer that allows the holotoxin to interact with a higher affinity to the ganglioside target and/or other proteins associated with the ganglioside target. The structure of the LT-IIc holotoxin shows that the A-subunit is attached to the B-pentamer via non-covalent forces that hold an alpha-helical A2-domain in the center of the B-pentamer’s ring structure, while the enzymatically active A1-domain is connected to the B-pentamer only through non-covalent interactions with the A2-domain [36], suggesting that any stabilizing effect the A-subunit exerts on the B-pentamer ring structure is modulated by the A2-domain. To address whether the A2-domain of LT-IIc is sufficient to recover the full cytotoxic activity on the B-pentamer, LT-IIc-A2-Flg, a chimeric protein in which the A1-subunit of LT-IIc holotoxin is replaced with a Flag epitope tag linked to the native A2-domain, was used to treat MDA-MB-231 cells to assess the contributions of the A2-domain to cytotoxicity (Figure 5A). MDA-MB-231 cells were treated in quadruplicate with 62 nM of either LT-IIc holotoxin, LT-IIc-A2-Flg, or the B-pentamer (LT-IIc-B5) for 48 h and the viability was measured by MTT assay and compared to untreated MDA-MB-231 cells. Figure 5B shows that exposure to LT-IIc holotoxin resulted in a 73% reduction in cell viability while exposure to LT-IIc-B5 resulted in only a 30% reduction in viable cell numbers. Treatment with LT-IIc-A2-Flg reduced the cell viability by 50%. Although the A2-domain improved the cytotoxic effect of the B-pentamer, the LT-IIc holotoxin was still significantly more cytotoxic than LT-IIc-A2-Flg, thus indicating that the A1-domain strongly assists in stimulating cell death in MDA-MB-231 cells. To address whether the A1-domain contributes to the ganglioside binding affinity, the capacity of LT-IIc-A2-Flg to compete for binding to MDA-MB-231 cells was examined. MDA-MB-231 cells were fixed and stained with AlexaFlour-488 labeled LT-IIc holotoxin in the presence or absence of equal concentrations of unlabeled LT-IIc holotoxin or LT-IIc-A2-Flg to determine, by flow cytometry, if either protein had a competitive advantage in regards to binding to gangliosides expressed on MDA-MB-231 cells. Both unlabeled LT-IIc holotoxin and unlabeled LT-IIc-A2-Flg similarly displaced AlexaFlour-488 labeled LT-IIc holotoxin, reducing the MFI to 51.0% +/− 4.2% and 48.8% +/− 2.4%, (Figure 5B), respectively. These binding data indicated that LT-IIc holotoxin and LT-IIc-A2-Flg exhibit equivalent binding affinities toward MDA-MB-231 cells. These data support the model that the A1-domain is critical for the full cytotoxic effectiveness of LT-IIc holotoxin.

### 2.8. Cytotoxicity Is Triggered by Ganglioside Ligation

The ganglioside-binding profile of LT-IIb was greatly altered by a single threonine (T) to isoleucine (I) substitution at amino acid position 13 in the B-subunit, which is conserved in all three type II HLTs [19]. This amino acid substitution in LT-IIb manifested in a diminished capacity to bind to NeuAc-gangliosides despite it maintaining an affinity for NeuGc-gangliosides [37]. To evaluate the effect of an altered ganglioside-binding profile on the enterotoxigenic properties of LT-IIc, a T13I mutant of LT-IIc was engineered [LT-IIc(T13I)].

**Figure 6:**
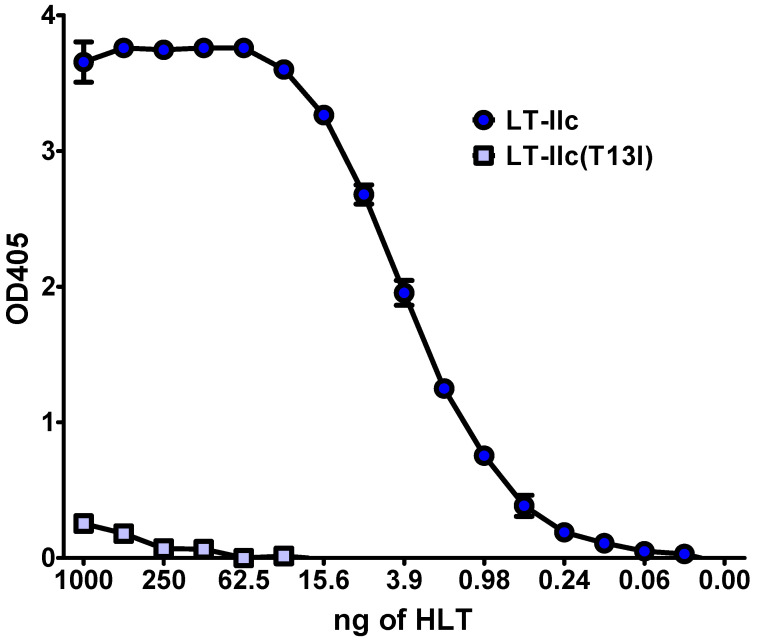
Effects of T13I substitution on LT-IIc binding affinity toward MDA-MB-231. MDA-MB-231 cells were fixed to a 96-well plate and treated, in duplicate, with two-fold dilutions of a 1 μg/mL solution of wt LT-IIc or LT-IIc(T13I). HLTs bound to MDA-MB-231 were detected by ELISA. Data points represent mean +/− SD.

To determine the effects of the T13I mutation on the ganglioside-binding properties of LT-IIc(T13I), MDA-MB-231 cells were fixed onto ELISA plates and probed with the mutant holotoxin (Figure 6). In comparison to wt LT-IIc, LT-IIc(T13I) exhibited a significantly lower binding affinity toward MDA-MB-231 cells. While wt LT-IIc binding was saturated at concentrations above 19.3 pM, binding of LT-IIc(T13I) was undetectable at concentrations below 31 nM. Despite this low affinity for MDA-MB-231, LT-IIc(T13I) induced intracellular accumulation of cAMP (Figure 4A). MDA-MB-231 treated with 62 nM of LT-IIc(T13I) for 6 h accumulated 33.9 pmol/mL cAMP, a statistically insignificant reduction from the 44.2 pmol/mL cAMP elicited by the high-binding-affinity wild-type LT-IIc, and significantly higher than untreated cells (*p* < 0.001) (Figure 4A). These data indicated that LT-IIc(T13I) not only bound to the cells, but was internalized. When the LT-IIc(T13I) was compared to wt LT-IIc, a distinct difference in the cytotoxic effect on TNBC cells was observed. Over 70% of cells were killed by wt LT-IIc at a concentration of 31 nM. LT-IIc(T13I), on the other hand, elicited only a negligible cytotoxic response on MDA-MB-231 cells even at concentrations of up to 250 nM, and the calculated IC50 for LT-IIc(T13I) was 466.1 nM (Figure 4B). These data strongly indicated that the ganglioside binding of LT-IIc, but not the HLT’s ADP-ribosyltransferase activity, or a capacity to increase intracellular cAMP, was critical for LT-IIc’s cytotoxic response for MDA-MB-231 cells.

### 2.9. Inhibiting Ganglioside Synthesis Rescues TNBC Cells from Cytotoxicity

**Figure 7:**
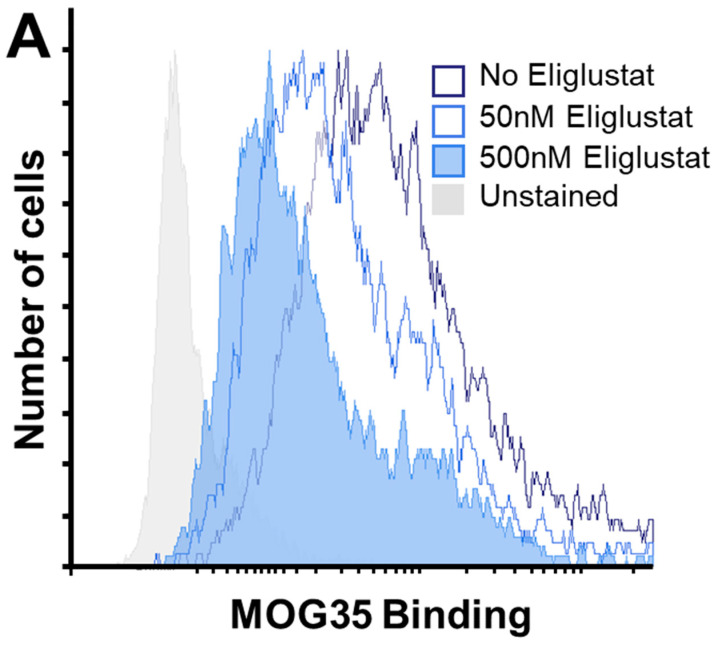

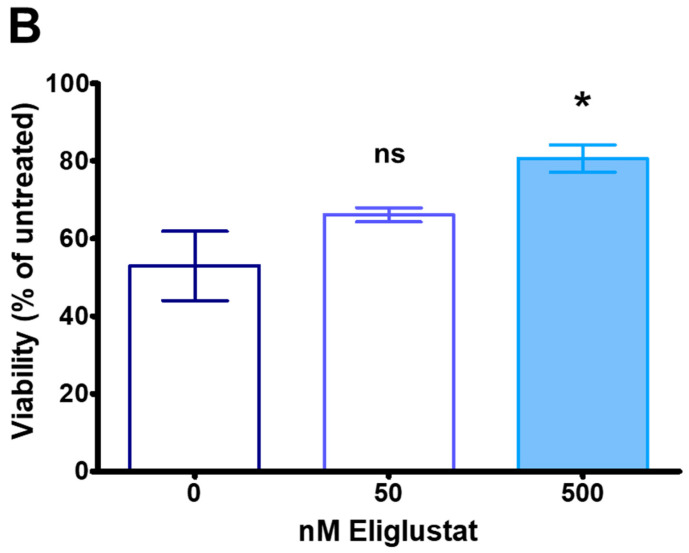
Effects of eliglustat on ganglioside GD1a expression- and LT-IIc-dependent cytotoxicity on MDA-MB-231 cells. MDA-MB-231 cells were cultured in the absence or presence of 50 nM or 500 nM eliglustat for 4 days prior to (**A**) analysis of MOG35 immunostained cells measured by flow cytometry and (**B**) treatment with 31 nM LT-IIc for 24 h prior to analysis of viability measured by MTT assay (n = 6). Viability is calculated as % of untreated cells cultured under same eliglustat concentration. Data points represent mean +/− SEM. Statistical significance was measured by comparison to LT-IIc-treated cells not cultured with eliglustat using one-way ANOVA. * *p* < 0.5; ns = no statistical significance.

To confirm that the binding of LT-IIc to gangliosides expressed on the surface of MDA-MB-231 cells was critical for initiating the cytotoxic response to LT-IIc, experiments were designed to determine if the LT-IIc-induced cytotoxicity was diminished or abrogated if the levels of gangliosides on the cell surface were pharmacologically reduced. Total glycosphingolipids isolated from MDA-MB-231 were found to contain ganglioside GD1a [38], a disialoganglioside for which LT-IIc has a strong binding affinity [22,39]. Eliglustat is a pharmacological inhibitor (IC50 = 25 nM) of glucosylceramide synthase, the enzyme that catalyzes the first committed step in subsequent ganglioside biosynthesis that includes GD1a [40]. A flow cytometric analysis of MDA-MB-231 cultured in the presence of eliglustat and stained with MOG35, a mAb with specificity for ganglioside GD1a [41], revealed that the surface expression of GD1a was reduced in a dose-dependent manner in those cells, consistent with the likely downregulation of all gangliosides, in general [40,42]. MDA-MB-231 cells cultured for 4 days with 50 nM or 500 nM of eliglustat and stained with MOG35 exhibited a decrease in mean fluorescent intensity (MFI) that was reduced by more than two-fold (12,492 MFI) and four-fold (6923 MFI) what was observed for untreated cells (28,071 MFI) (Figure 7A). Treatment with eliglustat alone had a significant impact on cells’ survival. MDA-MB-231 cells treated with 50 nM and 500 nM of eliglustat displayed a reduced cell viability of 87% and 60% compared to untreated cells, respectively (Figure S1). Subsequent cytotoxicity assays demonstrated that the 500 nM eliglustat-treated MDA-MB-231 cells were significantly less sensitive than control cells to LT-IIc cytotoxicity. MDA-MB-231 cells cultured for 4 days in the presence of 500 nM of eliglustat exhibited only a 19% decrease in viability when exposed to 31 nM LT-IIc for 24 h, significantly less than the 47% reduction observed for control cells (no eliglustat) treated with LT-IIc (Figure 7B). Collectively, these data further supported the model that LT-IIc triggers the irreversible cytotoxic response in MDA-MB-231 cells by ligating surface gangliosides without the need for ADP-ribosyltransferase activity or a capacity to increase intracellular cAMP.

## 3. Discussion

We have shown that, unlike other members of the HLT family, LT-IIc elicits a cytotoxic response that was specific for TNBC cell lines [29]. To extend these findings, we sought to determine if this cytotoxic effect remained relevant in primary TNBC cells from clinical specimens. Indeed, LT-IIc had a profound impact on the viability of primary cells that were isolated from resected human tumors of the TNBC subtype, while other HLTs exhibited little or no effect on the primary TNBC cells (Figure 1). These results support the initial findings [29] and motivate future experimentation to evaluate the clinical utility of LT-IIc as a therapeutic agent against TNBC. A pre-existing immune response against HLTs did not interfere with the HLTs’ ability to evoke an adjuvant response, thus suggesting that the LT-IIc could be used in multiple administrations without affecting its cytotoxic activity (Figure S2 and [43]). The uniqueness of the response of TNBC cells toward LT-IIc is intriguing, given that all HLTs possess similar functions of ADP-ribosyltransferase activity of the A1-domain and the B-pentamer’s ganglioside-binding affinity. Deciphering this feature of LT-IIc will determine the mechanism(s) behind the cytotoxic response in TNBC cells, as well as identifying the target that triggers the response.

Both LT-IIc and CT increased the intracellular concentration of cAMP in MDA-MB-231 to 50 nM after a 12 h exposure (Figure 2A). The levels of cAMP induced by LT-IIc, however, attenuated more rapidly than the cAMP levels induced by the treatment of cells with CT (Figure 2A), possibly due to the robust cytotoxic response elicited by LT-IIc. Treatment with either LT-IIc or CT also induced an increase in phosphorylated CREB (Figure 3). These results indicated that cAMP signaling is functional in this TNBC cell line and that both LT-IIc and CT had the capacity to stimulate the phosphorylation of a major cAMP-dependent transcriptional regulator. The fact that LT-IIc, but not CT, invoked a cytotoxic response suggested that cAMP signaling is not sufficient to initiate apoptosis and necroptosis in MDA-MB-231 cells (Figure 2B). cAMP targeting, using analogs such as dibutyryl-cAMP, 8-bromo-cAMP, and 8-chloro-cAMP, has been shown to suppress growth in various cell types by triggering apoptosis or cell-cycle arrest [30,31]. Yet, millimolar quantities of cAMP analogues were required to suppress TNBC cell growth due to the rapid efflux and decomposition of cAMP in that cell type [44].

A central dogma in the enterotoxin field is that the sole function of the A-subunit of type I and type II HLTs is to ADP-ribosylate the GSα subunit of the G protein regulatory complex to constitutively activate the cell’s adenyl cyclase and subsequently increase the concentration of intracellular cAMP. Thus, additional experiments were performed to evaluate the role of cAMP in cytotoxicity using LT-IIc(dblA), a site-specific mutant of LT-IIc, in which the prospective active site for ADP-ribosylation had been genetically interrupted. Biochemical assays confirmed that LT-IIc(dblA) was deficient in stimulating the accumulation of intracellular cAMP in MDA-MB-231 (Figure 4A). Using this mutant, it was shown that LT-IIc(dblA) exhibited a similar cytotoxicity profile as wt LT-IIc for MDA-MB-231 cells (Figure 4B). Similar substitutions in the type II family co-member LT-IIb eliminated the ability of this HLT to stimulate cellular infiltration into the immunization site of ID administered vaccine in mice [34], indicating that the mechanisms promoting cytotoxicity by LT-IIc are likely different from those involved in immune cell stimulation by either LT-IIc or LT-IIb. Clearly, these experiments demonstrated that the induction of cAMP by LT-IIc was dispensable for the cytotoxic activity of LT-IIc for MDA-MB-231 cells.

While it was clear that neither ADP-ribosylation nor the elevation of intracellular cAMP had a critical role in promoting the cytotoxicity of MDA-MB-231 by LT-IIc, it was feasible that the cytotoxic response induced by LT-IIc was triggered by some unknown function of the A polypeptide. The recombinant B-pentamers of both type I and type II HLTs, expressed in the absence of their respective A-subunit, assemble properly and retain the ganglioside binding properties of their holotoxin counterparts [35]. Although the B-pentamer of LT-IIc exhibited some degree of cytotoxicity for MDA-MB-231 cells, LT-IIc-B5 was less potent than the holotoxin. Additionally, LT-IIc-A2-Flg, which retains the A2-domain as a part of its toxin assembly, significantly improved the cytotoxic effect, although it was still less potent than the holotoxin (Figure 5B) despite exhibiting a similar binding affinity toward MDA-MB-231 cells (Figure 5C). These data suggested that the presence of the A-subunit had a role in imparting full cytotoxicity for LT-IIc (Figure 4B). The observation that full cytotoxic activity was observed using higher concentrations of LT-IIc-B5, however, indicated that the diminution of potency may be an indirect effect.

Nevertheless, it was evident that the cytotoxic effect of LT-IIc on MDA-MB-231 could still be elicited by LT-IIc-A2-Flg and, to a lesser degree, by LT-IIc-B5, even in the absence of part or all of the A-subunit, suggesting that any direct role of the A-subunit (e.g., ADP-ribosylation) is nonessential for the cytotoxic response. Overall, the data indicate that triggering cytotoxicity required only the capacity of LT-IIc to interact with cell-surface gangliosides.

LT-IIc has a distinctive ganglioside-binding profile that differs from the ganglioside-binding profiles of the other type I and type II HLTs [22]. Since cytotoxicity toward TNBC is unique to LT-IIc among the family of type I and type II HLTs, it was reasonable to conclude that this cytotoxic activity is due, at least in part, to the capacity of LT-IIc to bind to one or more gangliosides that are either uniquely or predominantly expressed on TNBC cells. Since LT-IIc has also been shown to be cytotoxic for TNBC cells isolated from primary human tumors (Figure 1), it is likely that this model of ganglioside expression extends beyond cultured TNBC cell lines. Prior studies on the immunology of the type II HLTs demonstrated [45,46] that an isoleucine for threonine substitution at amino acid position 13 in the B-pentamer of LT-IIb significantly altered the ganglioside-binding profile of that HLT [37,47,48]. A mutant of LT-IIc harboring this same amino acid substitution (LT-IIc(T13I)) exhibited a similar ability to stimulate cAMP production in MDA-MB-231 as wt LT-IIc (Figure 4A) despite a marked reduction in its ability to bind to MDA-MB-231 (Figure 6). When evaluated for cytotoxicity, LT-IIc(T13I) had a significantly reduced cytotoxic property even at higher concentrations (Figure 4B). Notably, the cytotoxic activity of LT-IIc(T13I) was essentially identical to the cytotoxic profile of CT, which has little capacity for killing TNBC cells [29] (Figure 4B).

To further establish that ganglioside ligation was essential for cytotoxicity, a pharmacological approach was taken. Eliglustat is a pharmacological inhibitor of glucosylceramide synthase, the enzyme that catalyzes the transfer of glucose to ceramide, which is the first committed step in glycolipid biosynthesis. Decreasing the total number of gangliosides expressed on the surface of eliglustat-treated MDA-MB-231 cells rescued the cells from LT-IIc cytotoxicity (Figure 7A,B). The inability of LT-IIc(T13I) to elicit a response in MDA-MB-231 and the capacity of eliglustat to reduce the cytotoxic response of LT-IIc firmly established that ganglioside binding was the initiating event for the capacity of LT-IIc to trigger cytotoxicity in MDA-MB-231 cells.

Gangliosides, which are expressed in all mammalian tissues, are comprised of a sialylated glycan attached to a ceramide lipid core [49]. Variations in the sites of insertion of these sialoglycans and the lipid components of ceramide combine to generate a diverse population of gangliosides (e.g., monosialyl-, disialyl-, and trisialyl-gangliosides). The roles of gangliosides in regulating cellular functions are not fully understood due to difficulties in analyzing and manipulating these molecules. Gangliosides, however, have been shown to modulate receptor signaling either by direct binding [50], by interaction with lipid rafts [51], or through intracellular trafficking [52]. There have been a number of reports on the expression of specific gangliosides in individual cancers, which includes the expression of ganglioside GD2 in breast cancer cells [38]. While the expression of disialyl gangliosides is correlated with malignant properties in various cancer systems [53,54,55], the expression of certain monosialyl gangliosides can suppress those properties [56,57,58]. LT-IIc interacts strongly with the monosialyl gangliosides GM1, GM2, and GM3, and with the disialyl ganglioside GD1a [22]. All four of these gangliosides have been identified in solvent extracts of MDA-MB-231 cells, which are sensitive to LT-IIc, and in MCF7, a non-TNBC cell line that is resistant to the cytotoxic activities of LT-IIc [29,59]. It is hypothesized that one or more types of gangliosides expressed on the surface of MDA-MB-231 cells are not expressed on the surface of MCF7 cells or on the surface of normal breast epithelial cells. Alternatively, the surrounding membrane environment, unique to the TNBC cell, may affect ganglioside’s function in triggering the cytotoxic response. For example, after binding to ganglioside GM1 and uptake by the cell, CT elicits ion efflux in human intestinal cells, which is not a lethal event. In those cells, the ganglioside GM1 is located in lipid rafts, which are sites for various signal transduction events. In contrast, LT-IIb, which binds to ganglioside GD1a, fails to elicit efflux in human intestinal cells since ganglioside GD1a does not localize to lipid rafts in human intestinal cells [60]. This observation elicits two intriguing questions: “Does LT-IIc participate in a cytotoxic-triggered signal transduction event that is initiated outside of lipid rafts?” “What is the ganglioside or gangliosides on TNBC that is bound by LT-IIc to initiate cytotoxic activity?” The initial step in resolving these questions is to not only identify the respective “cytotoxic” ganglioside(s) on MDA-MB-231 cells, but to examine the environment in which these gangliosides reside on the cell surface and what, if any, co-receptors cooperatively interact with those gangliosides. The capacity of eliglustat to reduce ganglioside expression on the surface of MDA-MB-231 cells and to render those cells less susceptible to LT-IIc makes it feasible to identify those “cytotoxic” gangliosides by use of ganglioside reconstitution experiments [61]. That pharmacological approach and other genetic approaches (iRNA and CRISPR/Cas9) are in process of being undertaken to identify the relevant gangliosides on MDA-MB-231 cells that are triggers for TNBC cytotoxicity.

## 4. Conclusions

The data presented herein suggest the unique or preferential presence of a ganglioside or a set of gangliosides located on the surface of MDA-MB-231 that, when bound by LT-IIc, initiates a series of cellular events that lead to cell death. The cytotoxic activity of LT-IIc is not dependent on its ADP-Ribosylase activity. Rather, these experiments revealed a novel mechanism by which ganglioside ligation by LT-IIc modulates a cytotoxic response in TNBC cells, but not in non-transformed cells. Furthermore, the development of humanized monoclonal antibodies or other biologics (such as LT-IIc) to target those gangliosides that will trigger that cytotoxic response in TNBC would revolutionize the treatment landscape for both primary and metastatic TNBC. Therefore, the identification of the ganglioside target(s) of LT-IIc and revelation of the downstream events that promote the LT-IIc-dependent cytotoxic response in TNBC cells will be crucial for the development of new and effective treatment options for individuals suffering from this aggressive breast cancer.

## 5. Methods

### 5.1. Cell Lines and Reagents

The breast cancer cell line MDA-MB-231 (CRM-HTB-26) was obtained from ATCC (Manassas, VA, USA). MDA-MB-231 cells were cultured in chemically defined Ultraculture medium (Lonza, Basel, Switzerland) supplemented with 20 mM L-glutamine and 1% penicillin/streptomycin. All media additives were purchased from Sigma-Aldrich (St. Louis, MO, USA) with the exception of basal media and 1% penicillin/streptomycin (Thermo-Fisher, Grand Island, NY, USA). Cells were cultured at 37 °C in the presence of 5% CO_2_. Eliglustat was obtained from Cayman Chemicals (Ann Arbor, MI, USA). Antibodies to CREB (86B10) and phospho-CREB (ser133) (8763) were purchased from Cell Signaling Technologies (Danvers, MA, USA). Mouse monoclonal Anti-D tag (G191) was purchased from ABM (Richmond, BC, Canada) and mAb Anti-GD1a (MOG35) was supplied by Dr. Hugh Williston [41]. MTT reagent thiazolyl blue tetrazolium bromide was purchased from Sigma-Aldrich.

### 5.2. Isolating and Culturing Primary Cells from Human, Resected Tumors

Tissue samples were provided by the Pathology Resource Network at Roswell Park Cancer Institute (Buffalo, NY, USA). After weighing, tissue samples were minced and resuspended in a solution of 1% collagenase/hyaluronidase (Stemcell Technologies, Cambridge, MA, USA) in complete Epicult-C media (Stemcell Technologies), supplemented with 2.4 mg/mL hydrocortisone, 25 μg/mL Insulin, 25 ng/mL epidermal growth factor, and 0.05 ng/mL cholera toxin (CT). Tissue digestion proceeded at 37 °C for 4–18 h. Primary cells were separated from the non-cellular components of tumor extracellular matrix by successive steps through 500 μm, 100 μm, and 40 μm mesh filters. Cells were washed with PBS containing 0.5 mM EDTA, resuspended in complete Epicult-C medium (Stemcell Technologies) supplemented with 2.4 mg/mL hydrocortisone, 25 μg/mL Insulin, 25 ng/mL epidermal growth factor, 0.05 ng/mL CT, and 5% FBS to facilitate cell attachment, and transferred to a T-25 flask. After 24 h, the medium, containing unattached cells, was removed and the attached cells were gently washed and replenished with medium containing no FBS to inhibit the growth of stromal cells. Cells were cultured at 37 °C, 5% CO_2_ and serum-free medium was replenished every three days.

### 5.3. Construction of LT-IIc(T13I), LT-IIc(dblA), LT-IIc-A2-Flg and LT-IIc-B5 Expression Plasmids

The operon that encodes the A- and B-subunits of LT-IIc was cloned into pBluescript SK II (+) (Stratagene, La Jolla, CA, USA) to generate pCB1 using primers designed to incorporate 6-histidine residues at the c-terminus of the B-polypeptide [22]. Complementary oligonucleotides 5′-GATAAATGCGCTTCGATTACGGCCAAGCTTGTAC-3′ and 5′-GTACAAGCTTGGCCGTAATCGAAGCGCATTTATC-3′ (Integrated DNA Technologies, Coralville, IA, USA) contain a nucleotide sequence designed to alter codon 13 of the LT-IIc B-polypeptide, resulting in a threonine to isoleucine substitution (T13I). A plasmid-encoding recombinant LT-IIc(T13I) was engineered using these oligonucleotides to amplify pCB1 by site-directed mutagenesis to produce plasmid pCB2. A 553 bp Geneblock (Integrated DNA Technologies) encoding the LT-IIc A polypeptide between the internal restriction enzyme sites *Sp*hI and *Eco*RI was synthesized containing a nucleotide sequence designed to alter codons 59 and 108 of the A polypeptide, producing serine to lysine (S59K) and a glutamic acid to lysine (E108K) substitutions. The Geneblock was digested with *Sph*I and *Eco*RI (ThermoFisher, Waltham, MA, USA) and the fragments ligated into pCB1 digested with the same enzymes to generate pCBGB. Oligonucleotides 5′-GGGTCGACATCGTTTGCTGAG-3′ and 5′-GGGTCGACTATAAGGACGATGACGATAAGGCATGGCGAGAGCTGC-3′ (Integrated DNA Technologies, Coralville, IA, USA), which contain a nucleotide sequence designed to incorporate a NYKDDDDK amino acid sequence between the A-subunit leader peptide and the A-2 domain, were used to amplify pCB1 by inverse PCR to generate pCB-Flg in order to express LT-IIc-A2-Flg, a chimeric protein in which the A1-domain is replaced with a Flag epitope tag. The plasmid-encoding LT-IIc-B5 (pJCH3.2) was previously engineered [21]. All plasmids generated were confirmed by nucleotide sequencing (Roswell Park Cancer Center, Buffalo, NY, USA).

### 5.4. HLT and B-Pentamer Expression and Purification

All plasmids containing recombinant HLTs or B-pentamers were introduced into *E. coli* DH5αF′Kan (Life Technologies, Inc., Gaithersburg, MD, USA) and expression was induced by addition of isopropyl-β-d-thiogalactoside to the culture medium. Recombinant HLT and B-pentamer were extracted from the periplasmic space and purified to homogeneity by nickel affinity chromatography and Sephacryl-100 gel filtration chromatography (VWR, Piscataway, NJ, USA) using an ÄKTA-FPLC (Pharmacia, Peapack, NJ, USA) [48].

### 5.5. cAMP ELISA

Intracellular cAMP accumulation was measured using a cAMP enzyme immunoassay kit (Cayman Chemical Co.). MDA-MB-231 cells were cultured overnight in 12-well plates at a density of 2.5 × 10^5^ cells/well and treated with 62 nM HLT or B-pentamer (equivalent to 5 μg/mL HLT or 3.4 μg/mL B-pentamer), in replicates of three, for the indicated time-points. After incubation, cells were extracted for 20 min at RT with 110 μL of 0.1 M HCl, scraped from the culture wells, centrifuged to clear the extracts of cell debris, diluted to 1:2 with ELISA buffer, and measured to obtain cAMP amounts according to manufacturer’s instructions. Untreated cells were employed as a negative control.

### 5.6. MTT Assay

Cell viability was measured using the MTT [3-(4,5-dimethylthiazol-2-yl)-2,5-diphenylterazolium bromide] tetrazolium reduction assay [62]. Briefly, MDA-MB-231 or primary TNBC cells were cultured overnight in 96-well plates at density of 1 × 10^4^ cells/well and treated with either 0, 15.5 nM, 31 nM, 62 nM, 124 nM, and 248 nM in the titration experiment or with 62 nM of HLT or B-pentamer for 24 or 48 h before the addition of the MTT reagent (0.5 mg/mL final concentration). The number of replicates for each treatment ranged from 4 to 6. Formazan crystals produced by viable cells were solubilized with a solution of 40% dimethylformamide, 16% sodium dodecyl sulfate, and 2% glacial acetic acid (pH 4.7), and the absorbance was measured at 570 nm using a VersaMax tunable microplate reader (Molecular Devices, San Jose, CA, USA) and SoftMax Pro software version 5.4 (MDS Analytical Technologies, San Jose, CA, USA).

### 5.7. Western Immunoblotting

MDA-MB-231 cells were cultured overnight in 12-well plates at a density of 2.5 × 10^5^ cells/well and were either untreated or treated, in triplicate, with 62 nM LT-IIc or CT for 1, 3, and 6 h. After washing twice in PBS, cells were lysed in NP40 buffer (50 mM Tris-HCl (pH 7.5), 150 mM NaCl, 0.5% NP-40, 1 mM DTT, 20 μM sodium orthovanadate, 0.5 mM phenylmethylsulfonylfluoride) containing HALT protease inhibitor cocktail (ThermoFisher). Protein concentrations of the lysates were determined using the Pierce BCA Microplate Protein Assay Kit (ThermoFisher). About 20 μg of protein per lane was loaded onto a 12% SDS-PAGE gel and resolved gels were transferred to PVDF, blocked with 5% non-fat dry milk in TBS-T (containing 0.05% Tween-20), and probed with 1:1000 rabbit-anti-phospho-CREB (P-CREB) and 1:10,000 goat-anti-rabbit-IgG-HRP (Southern Biotech, Birmingham, AL, USA). Membranes were stripped using a mild stripping buffer (0.2 M glycine, pH 2.5; 0.05% Tween-20) and re-blocked and re-probed with 1:1000 mouse-anti-CREB and 1:10,000 goat-anti-mouse-IgG-HRP (Southern Biotech). Chemiluminescence imaging was performed using a ChemiDoc Multi-function Imager (Bio-Rad, Hercules, CA, USA). Densitometry was performed using ImageJ software (NIH, Bethesda, MD, USA).

### 5.8. Competitive Binding Assay

MDA-MB-231 cells were grown to 80% confluency and detached by brief trypsin exposure (2 min), followed by washing with media, then twice with PBS. Suspended MDA-MB-231 cells were passed through a 35 µM nylon strainer then fixed with 2% formaldehyde. Fixed MDA-MB-231 cells (1 × 10^5^ per test) were resuspended in PBS and pretreated for 20 min at RT with the addition of either PBS, 124 nM unlabeled LT-IIc, or 124 nM unlabeled LT-IIc-A2-Flg prior to the addition of 124 nM of LT-IIc that was labeled with Alexa Fluor 488 using Life Tech Alexa Fluor Antibody labelling kit (ThermoFisher) according to manufacturer’s protocol. After an additional incubation for 20 min at RT, cells were washed twice in FACS buffer (PBS without Ca or Mg, 1 mM EDTA, 2% FCS) and resuspended in FACS Buffer prior to flow cytometric analysis on a BD FACS Celesta 3 laser flow cytometer (BD Biosciences, San Jose, CA, USA). The flow cytometry results were analyzed using FlowJo™ v10.8.2 Software (BD Life Sciences).

### 5.9. Whole Cell ELISA

MDA-MB-231 cells were cultured overnight in 96-well plates at a density of 1 × 10^4^ cells/well, rinsed with PBS, and fixed with 10% phosphate buffered formalin. Fixed cells were blocked with PBS containing 10% horse serum and treated with 1000 ng followed by 16 2-fold dilutions of LT-IIc or LT-IIc(T13I) in PBS containing 1% horse serum. After washing to remove unbound HLT, cells were treated with rabbit-anti-LT-IIb-IgG (1:2000) [48], an LT-IIc cross-reactive polyclonal antibody, for 1 h at RT and washed with PBS containing 1% horse serum to remove unbound antibodies. Cells were treated with phosphatase-conjugated goat anti-rabbit IgG (1:4000) (Southern Biotech) for 30 min at RT, which was washed and developed using 1 mg/mL solution of nitrophenyl phosphate (Amresco, Solon, OH, USA) diluted in diethanolamine buffer (100 mL of diethanolamine, 1 mM MgCl_2_, and sufficient deionized H_2_O to bring the volume to 1 L, pH 9.8). Absorbance was measured at 405 nm using a VersaMax tunable microplate reader (Molecular Devices) and SoftMax Pro software version 5.4 (MDS Analytical Technologies).

### 5.10. Ganglioside GD1a Staining

MDA-MB-231 cells were cultured in T-25 flasks for four days in the absence or presence of 50 nM or 500 nM eliglustat. Culture medium was replaced daily with fresh eliglustat. Cells were detached using cell dissociation buffer, then collected and washed in FACS buffer (PBS without Ca or Mg, 1 mM EDTA, 2% FCS, 0.01% NaN_3_). Cells (5.0 × 10^5^) were stained with 6.5 μg of mouse anti-GD1a antibody MOG35 (41) for 1 h at RT, then washed twice in FACS buffer followed by 0.5 μg of anti-mouse-IgG-APC (eBioscience, San Diego, CA, USA) for 30 min at RT. Stained cells were washed and resuspended in FACS buffer and analyzed by flow cytometry on a BD Fortessa 5 laser flow cytometer (BD Biosciences, San Jose, CA, USA).

### 5.11. Data and Material Availability

The unique reagents utilized and the datasets obtained during the current study will be made available by the corresponding author to other researchers on reasonable request.

### 5.12. Statistical Analysis

Data were analyzed using GraphPad Prism software version 5.03 (GraphPad Prism Inc., La Jolla, CA, USA). Statistical significance of the data was determined using Student’s *t*-test and one-way or two-way ANOVA, as appropriate.

## Data Availability

The datasets obtained during this study and any unique reagents developed therein will be made available to researchers by the corresponding author upon reasonable requests.

## Supplementary Materials

The following supporting information can be downloaded at: https://www.mdpi.com/article/10.3390/toxins16070311/s1, Figure S1: Effects of eliglustat on MDA-MB-231 cell viability; Figure S2: Prior administration of LT-IIb(T13I) does not inhibit its adjuvant activity.

## Abbreviations

ADP	adenosine diphosphate
BCG	Bacillus Calmette-Guerin
cAMP	cyclic adenosine monophosphate
CREB	cAMP Response Element-Binding Protein
CT	cholera toxin
ER	estrogen receptor
Gsα	Gs-alpha subunit
HER2/Neu/ErbB2	human epidermal growth factor receptor 2
HLT	heat-labile enterotoxins
MFI	mean fluorescent intensity
MTT	thiazolyl blue tetrazolium bromide
NeuAc	N-acetylneuraminic acid
NeuGc	N-glycolylneuraminic acid
PKA	protein kinase A
PR	progesterone receptor
TNBC	triple-negative breast cancer
