# Supplementary material for: A Novel Cytotoxic Mechanism for Triple-Negative Breast Cancer Cells Induced by the Type II Heat-Labile Enterotoxin LT-IIc through Ganglioside Ligation
Source: Toxins (Basel). 2024 Jul 11;16(7):311. doi: 10.3390/toxins16070311 (PMC11281474; doi:10.3390/toxins16070311)

Supplemental Figures:

Figure S1: Effects of eliglustat on MDA-MB-231 cell viability. MDA-MB-231 cells were cultured in the absence or presence of 50 or 500nM eliglustat for 4 days prior to analysis of viability measured by MTT assay (in replicates of 6). Statistical significance was measured by comparison to 0nM treated cells using Pairwise T-test. *** p < 0.0001.

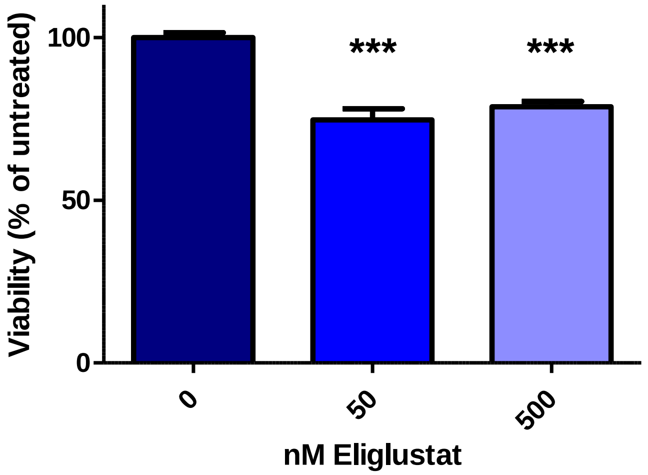

Figure S2: Prior administration of LT-IIb(T13I) does not inhibit its adjuvant activity. (A). Serum IgG against LT-IIb(T13I) from 4 groups of mice (4 mice/group) after administration with either PBS (1 and 2) or 1ug of LT-IIb(T13I) (3 and 4). (**B)**. Serum IgG against OVA from the same 4 groups of mice after administration of 50ug of OVA alone (1 and 3) or 50ug of OVA plus 1ug of LT-IIb(T13I) (2 and 4). **(C)**. Serum IgG against PspA from the same 4 groups of mice after administration of 10ug PspA alone (1) or 10ug PspA plus 1ug of LT-IIb(T13I) (2, 3 and 4). For each round of immunizations, mice received intranasal immunizations on days 1, 10 and 20. 2nd and 3rd rounds of immunizations began 37 and 101 days after the 1st, respectively. Serum samples were collected 28 days after each round and IgG antibodies against T-IIb(T13I, OVA and PspA were measured by ELISA.

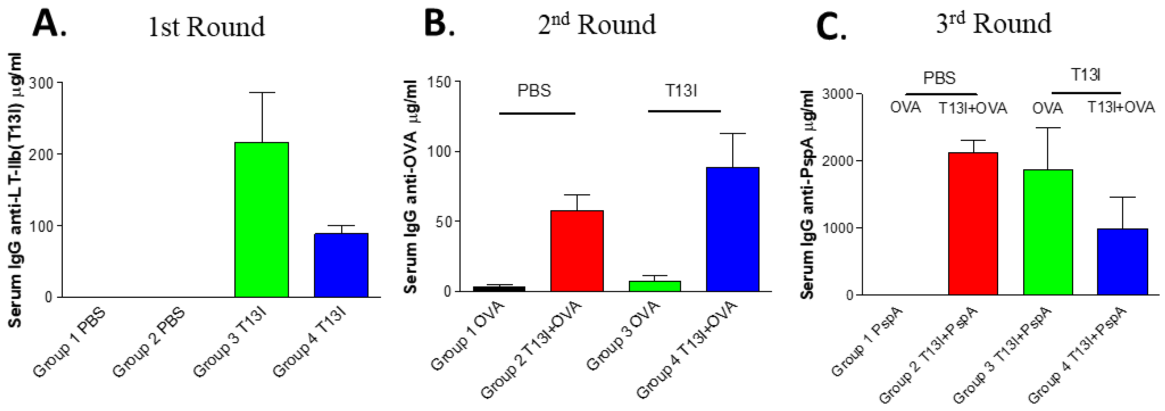

Supplement: Supplementary file 1 [file toxins-16-00311-s001.zip › toxins-2954729-supplementary.pdf]
